# Epidemiological and Clinical Profiles of Patients Leaving Against Medical Advice From the Pediatric Intensive Care Unit of a Tertiary Care Hospital: A Prospective Observational Study

**DOI:** 10.7759/cureus.96939

**Published:** 2025-11-16

**Authors:** Srishti Agarwal, Neeraj Anand, Sciddhartha Koonwar, SN Singh, Prabhaker Mishra

**Affiliations:** 1 Pediatrics, King George's Medical University, Lucknow, IND; 2 Biostatistics & Health Informatics, Sanjay Gandhi Post Graduate Institute of Medical Sciences, Lucknow, IND

**Keywords:** critical illness, india, leaving against medical advice, pediatric intensive care unit, socioeconomic factors

## Abstract

Background

Leaving against medical advice (LAMA) in pediatric intensive care units (PICUs) carries substantial risks, yet limited data exist from Indian settings. This study aimed to determine the incidence, demographic and clinical characteristics, and contributory factors for LAMA in a tertiary care PICU.

Methods

A prospective observational study was conducted at the PICU of King George’s Medical University, Lucknow, India, over 12 months. All children aged between one month and 12 years discharged against medical advice (DAMA) were included. Data on demographics, socioeconomic status, diagnosis, reasons for LAMA, and outcomes at 15 days were analyzed using IBM SPSS Statistics, version 23.0 (IBM Corp., Armonk, NY).

Results

Of 932 admissions, 135 (14.5%) left against medical advice. Among these patients, the mean age was 62.8 months; most were male, 76 (56.3%), and from rural areas, 78 (57.8%), and a total of 31 (23.0%) belonged to the lower socioeconomic class. The main reasons cited for LAMA were perceived poor survival, 85 (63.0%), and financial burden, 58 (43.0%). Parental illiteracy was reported in 27 (29.7%), and low income also showed significant associations for mortality among patients who left against medical advice.

Conclusion

LAMA rates in this PICU were high, predominantly driven by socioeconomic hardship and pessimism regarding prognosis. Interventions combining financial assistance, strengthened caregiver communication, and structured support services may help reduce LAMA and improve outcomes in critically ill children.

## Introduction

Leaving against medical advice (LAMA), also known as discharge against medical advice (DAMA), is a significant challenge in healthcare worldwide. It occurs when patients or guardians choose discharge before the treating physician’s recommendation. In pediatric settings, decisions are made by caregivers, often under stress and with limited understanding of the clinical consequences [1,2].

In pediatric intensive care units (PICUs), where patients are critically ill and require intensive support, LAMA can interrupt life-saving treatments, worsen illness, and increase mortality, creating ethical and professional dilemmas for providers [3]. While LAMA among adults is well studied, LAMA among children, especially from PICUs, has received little attention. Reported incidence ranges from 1.2% in Nigeria to 31.7% in other African nations, with Indian studies showing rates between 2.9% and 10.7% [4-7].

Decisions on LAMA are driven by multiple factors, including financial constraints, perceived poor prognosis, dissatisfaction with services, illiteracy, rural residence, and family obligations [8]. In a North Indian tertiary hospital, 41.8% of parents cited a hopeless prognosis, and 28.2% cited financial difficulty [6]. In developing countries, there is high mortality among PICU LAMA cases, particularly those on mechanical ventilation [9].

In India, lack of universal health coverage, overcrowded public hospitals, and suboptimal patient-provider communication further exacerbate the issue. Despite governmental child health initiatives, LAMA remains a major barrier to optimal pediatric outcomes. Notably, PICUs contribute a substantial proportion of pediatric LAMA cases, yet specific literature from India is almost nonexistent, and global studies remain limited [8,9].

This study aimed to determine the incidence of LAMA among children admitted to a tertiary care PICU in North India and to assess associated epidemiological and clinical factors.

## Materials and methods

Study design and setting

This prospective observational study was conducted in the PICU of the Department of Pediatrics, King George’s Medical University, Lucknow, Uttar Pradesh, India. The PICU is a 20-bed tertiary-level facility equipped with invasive and noninvasive ventilation, multiparameter monitoring, inotropic support, continuous renal replacement therapy, and other advanced life-support modalities. It serves as a referral center for critically ill pediatric patients from both rural and urban regions of Uttar Pradesh and neighboring states. The study was carried out over a period of 12 months, from December 2023 to December 2024, with 10 months dedicated to active data collection. The initial two months were used for finalizing the protocol, training the PICU staff in study procedures, and pilot testing the data collection tools. Ethical approval was obtained from the Institutional Ethics Committee (reference number: XXII-PGTSC-IIA/P50) before initiating the study.

Study population

The study population comprised all children between the ages of one month and 12 years admitted to the PICU during the study period. Eligibility for inclusion required that the child’s parents or legal guardians opt for DAMA and provide written informed consent for participation. Children were excluded if consent was not granted or if their medical records lacked sufficient clinical or discharge details. During the study period, 932 children were admitted to the PICU, of whom 135 (14.5%) met the inclusion criteria and were enrolled for analysis.

Operational definition of LAMA

For the purposes of this study, LAMA was defined as the voluntary withdrawal of a child from the PICU by a parent or legal guardian before the treating pediatric intensivist deemed the child fit for discharge, regardless of the patient’s clinical stability or ongoing need for critical care.

Data collection

Data collection was carried out prospectively at the time of DAMA using a pre-tested structured proforma (Appendix A). This proforma was developed following a review of existing literature on LAMA and pediatric critical care and was validated for content by two pediatric intensivists and a biostatistician. The content validity of the proforma was quantified using the Content Validity Index (CVI), with an Item-Level CVI (I-CVI) ranging from 0.83 to 1.00 and a scale-level CVI/average (S-CVI/Ave) of 0.92, indicating excellent content validity. The information recorded included demographic characteristics such as age, sex, birth order, family size, place of residence, and distance from the hospital. Socioeconomic status was determined using the Modified Kuppuswamy Scale (2024 revision) [10], which incorporates the education and occupation of the head of the family along with monthly household income. Clinical details included the primary diagnosis, associated comorbidities, duration of illness prior to hospital admission, Pediatric Index of Mortality 2 (PIM2) score at admission [11], length of PICU stay, and the need for advanced interventions such as mechanical ventilation, inotropic support, or dialysis.

The reasons for LAMA were documented verbatim from the caregivers during an in-person interview conducted at the time of discharge. These narratives were subsequently coded into categories, such as financial constraints, perceived poor prognosis, dissatisfaction with hospital services, cultural or religious beliefs, preference for home care, preference for alternative medicine, and other miscellaneous reasons.

Post-discharge outcomes were assessed through telephonic follow-up at seven and 15 days. The status at follow-up was classified as survival without readmission, survival with readmission, death after discharge, or loss to follow-up. A case was considered lost to follow-up if the caregivers could not be contacted despite three call attempts made at different times of the day.

Outcome measures

The primary outcome of interest was the incidence of LAMA among all PICU admissions during the study period. Secondary outcomes included the demographic and clinical profile of children who were LAMA and their short-term outcomes within 15 days of discharge.

Statistical analysis

All collected data were entered into Microsoft Excel (Microsoft Corp., Redmond, WA) using a double-entry method to minimize errors and were analyzed using IBM SPSS Statistics for Windows, Version 23.0 (IBM Corp., Armonk, NY) on the Windows 10 operating system (Microsoft Corp.). The distribution of continuous variables was assessed for normality using the Shapiro-Wilk test. Normally distributed data were presented as mean ± standard deviation (SD). Categorical variables were expressed as frequencies and percentages. Group comparisons for categorical variables were performed using the chi-square test or Fisher’s exact test where appropriate. Continuous variables were compared using the independent t-test for normally distributed data. Before conducting multivariable logistic regression, key assumptions such as the absence of multicollinearity and linearity of log-odds were verified. Model fit was evaluated using the Hosmer-Lemeshow goodness-of-fit test (p = 0.42), indicating an adequate fit of the model. To identify independent predictors of LAMA, multivariate logistic regression analysis was performed, including variables with a p-value <0.200 from univariate analysis. Adjusted odds ratios (aOR) with 95% confidence intervals (CI) were reported. All tests were two-tailed, and a p-value of less than 0.05 was considered statistically significant.

## Results

A total of 932 children were admitted to the PICU during the study period, of whom 135 (14.5%) were DAMA and 797 (85.5%) completed treatment (non-LAMA). The mean age of patients was comparable between groups (p = 0.615), with no significant difference in gender (p = 0.374) or residence (p = 0.311). Father’s education showed a strong association with LAMA status (p < 0.001), with illiteracy (30, 22.2%) and primary-level education (28, 20.7%) being more frequent among LAMA cases compared with non-LAMA patients. Notably, a higher proportion of children from the lowest socioeconomic stratum (31, 23.0%) left against medical advice compared to non-LAMA cases (31, 3.9%). LAMA families also reported a lower mean monthly income (Rs 7,237 ± 2,200) than non-LAMA families (Rs 7,856 ± 2,180; p < 0.001), and a greater proportion belonged to lower socioeconomic classes (p < 0.001). Other clinical characteristics, including Pediatric Index of Mortality 2 (PIM-2) score and predicted mortality risk, did not differ significantly between the two groups (p = 0.053 and p = 0.671, respectively). Distribution of major diagnostic categories also revealed group differences, with respiratory, central nervous system, and cardiac conditions more prevalent among LAMA cases (Table 1).

**Table 1:** Demographic and clinical characteristics of LAMA and non-LAMA patients (N = 932) * Socioeconomic status was determined using the Modified Kuppuswamy Scale (2024 revision) [10]. LAMA: leaving against medical advice; PIM-2: Pediatric Index of Mortality 2 [11]; CNS: central nervous system

Variable	Total (N=932)	LAMA (n=135)	Non-LAMA (n=797)	p-value
	Frequency (%)/Mean ± SD			
Age (in months)	61.0 ± 28.5	62.8 ± 29.4	60.7 ± 28.2	0.615
Gender				
Male	557 (59.8)	76 (56.3)	481 (60.4)	0.374
Female	375 (40.2)	59 (43.7)	316 (39.6)	
Residence				
Rural	501 (55.8)	78 (57.8)	423 (53.1)	0.311
Urban	431 (44.2)	57 (42.2)	374 (46.9)	
Father’s education				
Graduate	37 (4.0)	8 (5.9)	29 (3.6)	<0.001
Intermediate	126 (13.5)	20 (14.8)	106 (13.3)	
High school	257 (27.6)	25 (18.5)	232 (29.1)	
Middle school	290 (31.1)	24 (17.8)	266 (33.4)	
Primary	162 (17.4)	28 (20.7)	134 (16.8)	
Illiterate	60 (6.4)	30 (22.2)	30 (3.8)	
Socioeconomic status				
Upper	1 (0.1)	0 (0.0)	1 (0.1)	<0.001
Upper-middle	43 (4.6)	7 (5.2)	36 (4.5)	
Lower-middle	361 (38.7)	35 (25.9)	326 (40.9)	
Upper-lower	465 (49.9)	62 (45.9)	403 (50.6)	
Lower	62 (6.7)	31 (23.0)	31 (3.9)	
Family monthly income (Rs)	7,767 ± 2,150	7,237 ± 2,200	7,856 ± 2,180	<0.001
PIM-2 score	27.8 ± 15.4	31.5 ± 17.2	27.1 ± 14.9	0.053
PIM-2 mortality risk (%)	99.1 ± 1.2	99.7 ± 0.6	99.0 ± 1.3	0.671
Diagnosis group				
Infections/Sepsis/Poisoning	354 (38.0)	46 (34.1)	308 (38.7)	<0.001
Respiratory/CNS/Heart	282 (30.2)	53 (39.3)	229 (28.7)	
Hematology–oncology	149 (16.0)	23 (17.0)	126 (15.8)	
Multiple diseases	147 (15.8)	13 (9.6)	134 (16.8)	

**Figure 1:**
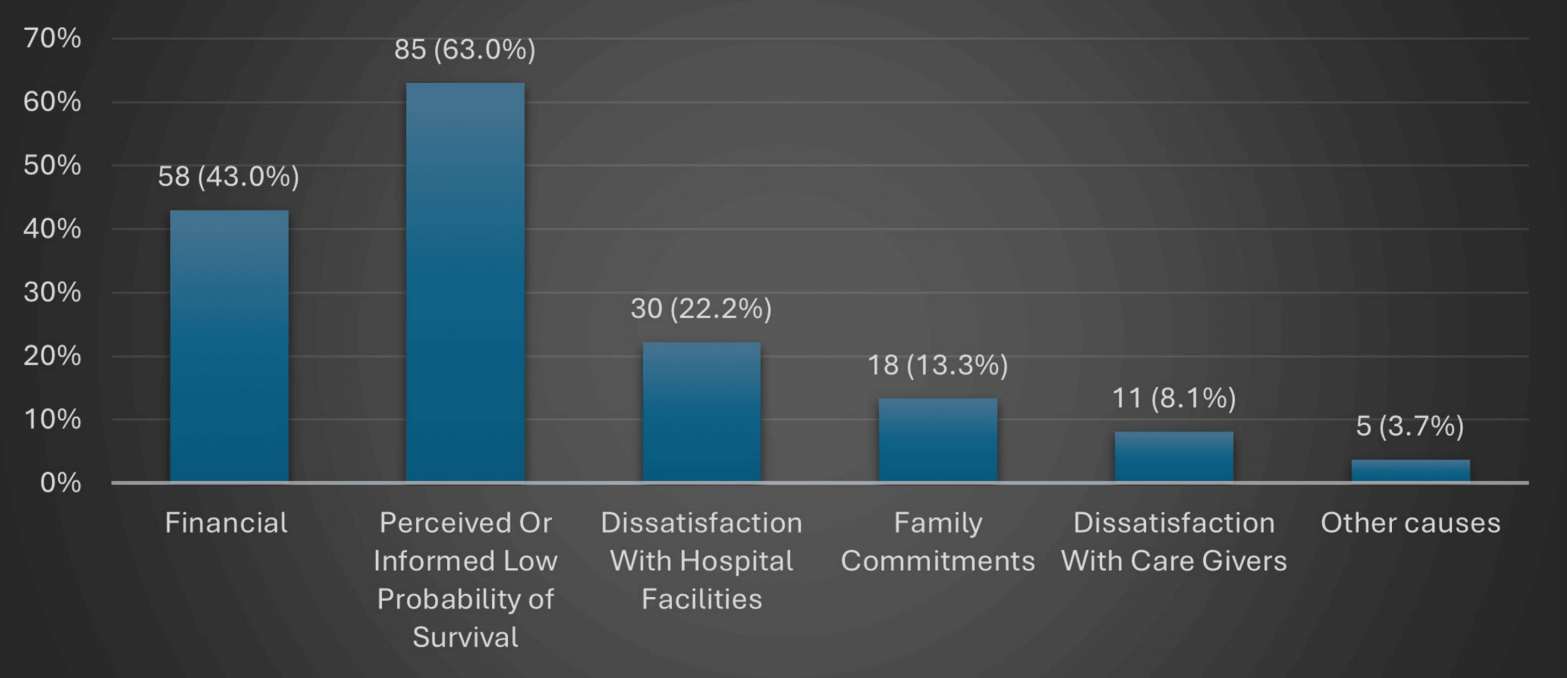
Reasons for opting for LAMA Multiple responses were permitted; hence, total percentages may exceed 100%. LAMA: leaving against medical advice

The most common reasons cited for LAMA were perceived poor survival chances in 85 (63.0%) children and financial burden in 58 (43.0%), followed by dissatisfaction with facilities in 30 (22.2%) and family obligations in 18 (13.3%) (Figure 1).

Mortality was significantly higher in children from rural areas (p = 0.044), from lower socioeconomic classes (p = 0.010), and in those who were moribund at admission (p < 0.001) or whose condition had deteriorated at the time of LAMA discharge (p < 0.001). In contrast, gender, religion, family type, decision-maker identity, father’s education, and father’s occupation did not show statistically significant differences between survivors and non-survivors. Among children who were DAMA, outcomes varied significantly with their condition at the time of discharge. Children discharged while in improved condition had a markedly higher survival rate (39, 88.6%), compared to those whose condition was unchanged (five, 11.4%) or deteriorated (0, 0.0%). All moribund patients at admission died within the follow-up period (Table 2).

**Table 2:** Characteristics of LAMA patients based on the outcomes (N = 135) * Socioeconomic status was determined using the Modified Kuppuswamy Scale (2022 revision) [10]. LAMA: leaving against medical advice

Variable	Total (n=135)	Death (n=91)	Alive (n=44)	p-value
	Frequency (%)			
Gender				
Male	76 (56.3)	46 (50.5)	30 (68.2)	0.053
Female	59 (43.7)	45 (49.5)	14 (31.8)	
Residence				
Rural	78 (57.8)	58 (63.7)	20 (45.5)	0.044
Urban	57 (42.2)	33 (36.3)	24 (54.5)	
Religion				
Hindu	103 (76.3)	65 (71.4)	38 (86.4)	0.056
Muslim	32 (23.7)	26 (28.6)	6 (13.6)	
Socioeconomic status*				
Upper-middle	7 (5.2)	3 (3.3)	4 (9.1)	0.01
Lower-middle	35 (25.9)	20 (22.0)	15 (34.1)	
Upper-lower	62 (45.9)	40 (44.0)	22 (50.0)	
Lower	31 (23.0)	28 (30.8)	3 (6.8)	
Type of family				
Joint	49 (36.3)	32 (35.2)	17 (38.6)	0.694
Nuclear	86 (63.7)	59 (64.8)	27 (61.4)	
Educational level of the father				
Graduate	8 (5.9)	4 (4.4)	4 (9.1)	0.066
Intermediate	20 (14.8)	11 (12.1)	9 (20.5)	
High school	25 (18.5)	15 (16.5)	10 (22.7)	
Middle school	24 (17.8)	15 (16.5)	9 (20.5)	
Primary school	28 (20.7)	19 (20.9)	9 (20.5)	
Illiterate	30 (22.2)	27 (29.7)	3 (6.8)	
Profession of the father				
Professional	4 (3.0)	2 (2.2)	2 (4.5)	0.231
Technicians and associate professionals	3 (2.2)	1 (1.1)	2 (4.5)	
Clerk/Farm	5 (3.7)	3 (3.3)	2 (4.5)	
Skilled worker	34 (25.2)	19 (20.9)	15 (34.1)	
Craft and related trade worker	14 (10.4)	8 (8.8)	6 (13.6)	
Plant and machine operator and assembler	18 (13.3)	14 (15.4)	4 (9.1)	
Elementary occupation	57 (42.2)	44 (48.4)	13 (29.5)	
Decision taken by				
Father	89 (65.9)	61 (67.0)	28 (63.6)	0.825
Mother	5 (3.7)	4 (4.4)	1 (2.3)	
Both	34 (25.2)	21 (23.1)	13 (29.5)	
Other	7 (5.1)	4 (4.4)	2 (4.5)	
Condition at admission				
Moribund	32 (23.7)	32 (35.2)	0 (0.0)	<0.001
Serious	103 (76.3)	59 (64.8)	44 (100.0)	
Condition at LAMA				
Deteriorated	57 (42.2)	57 (62.6)	0 (0.0)	<0.001
Improved	40 (29.6)	1 (1.1)	39 (88.6)	
Same	38 (28.2)	33 (36.3)	5 (11.4)	

Multivariable logistic regression analysis identified educational, socioeconomic, diagnostic, and clinical factors as significant independent predictors of LAMA. Fathers with middle- to below-graduate education (six to 12 years of schooling) had significantly lower odds of LAMA (aOR = 0.42; 95% CI: 0.18-0.95; p = 0.038) compared to those with graduate-level education and above. In contrast, children whose fathers were primary-educated or illiterate (0 to five years) had a higher, though nonsignificant, likelihood of LAMA (aOR = 1.63; 95% CI: 0.69-3.84; p = 0.266). Children from the lower socioeconomic class were almost three times more likely to LAMA (aOR = 2.78; p = 0.011) than those from the upper or middle classes. Diagnostic category was also strongly associated with LAMA-patients with respiratory, CNS, or cardiac illnesses had 7.72 times higher odds of LAMA (p < 0.001) compared to those with infections, sepsis, or poisoning, while hematology-oncology (aOR = 4.30; p = 0.003) and multiple-disease cases (aOR = 5.96; p < 0.001) were similarly at increased risk. When clinical prognosis variables were incorporated, children who were moribund or deteriorating at the time of LAMA had 6.84 times higher odds of leaving (95% CI: 2.92-15.99; p < 0.001), indicating that worsening clinical status substantially influenced the decision to discontinue care. Conversely, those who had improved before LAMA were significantly less likely to die subsequently (aOR = 0.28; 95% CI: 0.09-0.86; p = 0.027). In contrast, non-LAMA patients generally continued treatment and survived to discharge, emphasizing that LAMA was closely associated with an unfavorable prognosis. Model diagnostics demonstrated good fit (Hosmer-Lemeshow p = 0.42) with acceptable multicollinearity (variance inflation factor (VIF) < 2), confirming the robustness of the regression model (Table 3).

**Table 3:** Independent predictors of LAMA (N = 932) Socioeconomic status was determined using the Modified Kuppuswamy Scale (2024 revision). CI: confidence interval; CNS: central nervous system; LAMA: leaving against medical advice

Variable	Adjusted odds ratio (aOR)	95% CI (Lower–Upper)	p-value
Father’s education			
Graduate and above	Reference	—	—
Middle to graduate (6–12 years)	0.42	0.18–0.95	0.038
Primary to Illiterate (0–5 years)	1.63	0.69–3.84	0.266
Socioeconomic status			
Upper	Reference	—	—
Upper middle to Upper lower	0.54	0.22–1.32	0.174
Lower	2.78	1.26–6.14	0.011
Diagnosis group			
Infections/Sepsis/Poisoning	Reference	—	—
Respiratory/CNS/Heart	7.72	3.39–17.57	< 0.001
Hematology–oncology	4.3	1.65–11.22	0.003
Multiple diseases	5.96	2.55–13.95	< 0.001
Clinical prognosis variables			
Moribund/Deteriorating at LAMA	6.84	2.92–15.99	< 0.001
Improved before LAMA	0.28	0.09–0.86	0.027

## Discussion

This study assessed the incidence, epidemiological characteristics, and clinical profiles of pediatric patients who left against medical advice from a tertiary care PICU over a one-year period. The incidence of LAMA in our PICU was 135 (14.5%) out of 932 admissions, which is notably higher than most previously reported Indian studies by Awasthi et al., Roy et al., Datta et al., and Jha et al., where rates ranged from 2.9% to 10.7% [6,7,12,13]. Comparable rates have been reported in other low- and middle-income countries (LMICs), such as 13% in the Philippines by Macrohon et al., 15% in Nigeria by Jimoh et al., up to 19% in Pakistan by Somasetia, and 24% in Nepal by Basnet et al., suggesting that our findings reflect a persistent challenge in resource-limited pediatric critical care settings [1,9,14,15]. This higher rate may also reflect increased care complexity, limited financial protection, and the high proportion of families from lower socioeconomic backgrounds in our catchment area [16].

Demographically, the age and gender distribution of LAMA patients in our cohort was comparable to non-LAMA patients, and no significant difference in rural-urban residence was observed. In the present study, a greater proportion of LAMA cases were male and under five years of age. This reflects the general demographic distribution of admissions to pediatric intensive care units, where younger children and boys are more frequently hospitalized due to higher vulnerability to infections and acute illnesses. Similar demographic patterns have been reported in studies from South Asia and Africa, including those by Macrohon et al., Okoromah et al., Jimoh et al., Opara et al., and Hasan et al. [1,4,14,17,18]. Therefore, the male and under-five predominance among LAMA cases in our study likely mirrors the overall admission pattern rather than representing a specific risk factor for LAMA. In some contexts, male predominance has been explained by socio-cultural norms in India in the studies by Awasthi et al. and Kumar et al., where male children may initially receive more aggressive care but also face early withdrawal if the prognosis is perceived as poor due to financial or emotional constraints [6,19].

Socioeconomic and educational factors emerged as strong determinants of LAMA in our study. Nearly half of the LAMA patients belonged to the lower socioeconomic class, and the monthly family income was significantly lower among LAMA than non-LAMA patients. These results are in agreement with Awasthi et al., Roy et al., and Parasher et al., who all identified low income as a consistent driver of early DAMA [6,7,20]. Our use of the Modified Kuppuswamy Scale [10] confirmed that socioeconomic deprivation was strongly associated with LAMA. Macrohon et al., in the Philippines, Sharma et al., in India, and Duru et al., in Nigeria, similarly found that financial constraints were the second most common reason for LAMA, often intertwined with pessimism about clinical recovery [1,21,22].

Father’s education was also an important correlate, with illiteracy and only primary schooling being overrepresented among LAMA cases. Although our multivariable model showed that middle- to below-graduate-level education (six to 12 years) was paradoxically protective against LAMA compared to graduate-level education, illiteracy still carried a higher (albeit statistically non-significant) risk. This unusual pattern may reflect complex socio-cultural and economic interactions in our region, where more educated fathers may also have higher expectations from treatment and thus may be more likely to withdraw if outcomes seem unsatisfactory [23,24]. Other LMIC studies, such as those by Somasetia et al. and Abbas et al., have linked low health literacy and poor physician-family communication to premature withdrawal of critically ill children [9,25].

Clinical severity and diagnostic profile also influenced LAMA decisions. PIM-2 scores and predicted mortality risk did not differ significantly between LAMA and non-LAMA groups, suggesting that prognostic models alone cannot explain caregiver decisions. However, diagnosis type was a strong predictor: children with respiratory, central nervous system, or cardiac diseases had 7.72 times higher odds of LAMA; those with hematology-oncology diagnoses had 4.3 times higher odds; and those with multiple comorbid conditions had 5.96 times higher odds compared to children with infections, sepsis, or poisoning. Previous studies by Okoromah et al., Roy et al., and Sharma et al. have suggested that caregivers often perceive chronic, disabling, or relapsing conditions as less amenable to cure, especially when prolonged ICU stays or high-cost interventions are required [4,7,21]. Okoromah et al. and Kalraiya et al. reported similar trends in Nigeria and India, where neurologic and oncologic illnesses carried a higher likelihood of LAMA [4,26].

The most common reasons cited for LAMA were perceived poor survival chances in 85 (63.0%) children and financial burden in 58 (43.0%), followed by dissatisfaction with facilities in 30 (22.2%) and family obligations in 18 (13.3%). These reasons mirror findings from Awasthi et al. and Dutta et al., who noted that cultural acceptance of death at home, desire to avoid prolonged suffering, and cost-related distress often drive families to opt for LAMA [6,12]. Importantly, our data show that financial burden remains a major factor despite government-subsidized healthcare, likely due to out-of-pocket expenses for medicines and diagnostics, as well as indirect costs such as travel and lost income reported in previous studies by Datta et al. and Parasher et al. [12, 20].

When compared with non-LAMA patients, children who left against medical advice exhibited markedly poorer prognostic outcomes. Mortality was observed in 91 (67.4%) LAMA cases, whereas survival was notably higher among those discharged in improved condition (39, 88.6%). Logistic regression analysis further demonstrated that being moribund or deteriorating at the time of LAMA was a strong independent predictor of mortality (aOR = 6.84; 95% CI: 2.92-15.99; p < 0.001), whereas improvement before LAMA was protective (aOR = 0.28; 95% CI: 0.09-0.86; p = 0.027). These findings are consistent with earlier reports by Somasetia et al. (Indonesia) and Jimoh et al. (Nigeria), where critically ill pediatric patients who chose to leave against medical advice, particularly those on ventilatory support, experienced adverse outcomes [9,14].

Our study highlights significant gaps in caregiver counselling, financial preparedness, and continuity of care among critically ill children who left against medical advice [27,28]. Notably, children who were moribund at admission or deteriorating at the time of LAMA had near-universal mortality, whereas those discharged in improved condition showed higher survival [29]. These findings emphasize the need for timely counselling regarding prognosis, close monitoring of critically ill children, and targeted support for families facing financial and logistical challenges at the point of LAMA [30].

Limitations

Limitations of our study include its single-center design, which may limit generalizability, and reliance on caregiver self-report for reasons of LAMA, which may be affected by recall or social desirability bias. Additionally, follow-up for post-LAMA mortality was limited, and outcomes may be underestimated in some cases. Nonetheless, this study adds to the sparse literature on LAMA in PICU settings in India and highlights actionable targets for reducing premature withdrawal of critically ill children.

## Conclusions

This study highlights the concerning frequency of LAMA among pediatric patients in the PICU, often involving critically ill children with conditions such as central nervous system disorders and hematological malignancies, many of whom required ventilatory support. The decision to leave prematurely was influenced by a combination of low socioeconomic status, parental illiteracy, and perceived poor prognosis. Addressing this issue requires early, empathetic counselling of caregivers, with active involvement of social workers, psychologists, and financial counsellors. Strengthening access to financial assistance programs and government schemes, alongside improving parental understanding of disease outcomes, can help mitigate premature discharges. Implementing hospital protocols to identify at-risk families may further improve clinical outcomes.

## Figures and Tables

**Figure 2:**
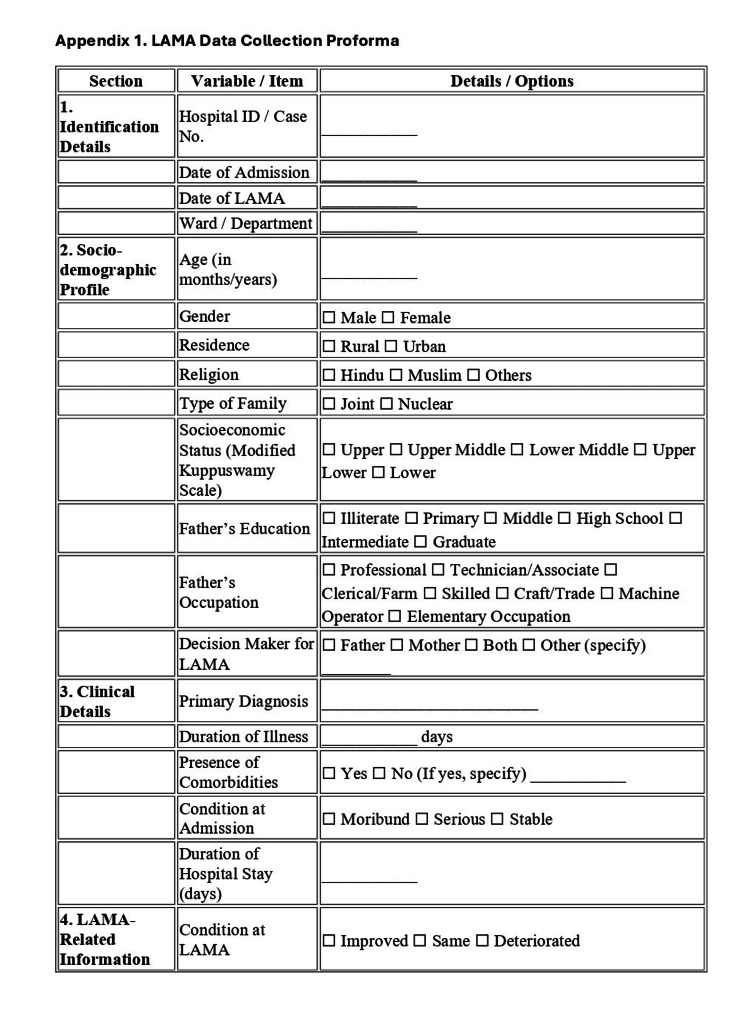
Proforma used for data collection: part 1

**Figure 3:**
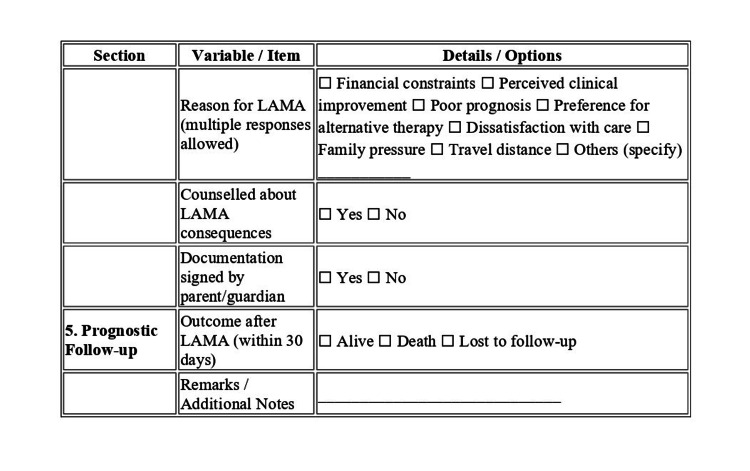
Proforma used for data collection: part 2

## Appendices

Appendix A
